# Clinical, Histopathological, and AS-OCT Correlations in Corneal Transplantation: Implications for Surgical Decision-Making

**DOI:** 10.22336/rjo.2026.55

**Published:** 2026

**Authors:** Ancuța-Georgiana Onofrei, Alina Gabriela Gheorghe, Ana-Maria Arghirescu, Ana Maria Dascălu

**Affiliations:** 1 Doctoral School, “Carol Davila” University of Medicine and Pharmacy, Bucharest, Romania; 2 BINE Ophthalmology Clinic, Bucharest, Romania; 3 Department of Ophthalmology, “Prof. Dr. Mircea Olteanu” Clinical Institute of Ophthalmological Emergencies, Bucharest, Romania; 4 “Carol Davila” University of Medicine and Pharmacy, Faculty of Medicine, Bucharest, Romania; 5 Ophthalmology Department, Emergency University Hospital, Bucharest, Romania

**Keywords:** corneal transplantation, anterior segment optical coherence tomography, histopathology, surgical technique selection, AS-OCT = anterior segment optical coherence tomography, DALK = deep anterior lamellar keratoplasty, DMEK = Descemet membrane endothelial keratoplasty, FECD = Fuchs endothelial corneal dystrophy, PK = penetrating keratoplasty

## Abstract

**Objective:**

*To evaluate the distribution of indications and surgical techniques in corneal transplantation and to describe the clinical, histopathological, and imaging correlations relevant to surgical technique selection.*

**Methods:**

A descriptive analysis of 233 corneal transplants performed between January 2021 and January 2026 was conducted. We recorded the indication for transplantation and surgical technique. In representative cases of pseudophakic bullous keratopathy, Fuchs endothelial corneal dystrophy (FECD), keratoconus, and fungal keratitis, we correlated slit-lamp biomicroscopy, histopathological findings, and anterior segment optical coherence tomography (AS-OCT).

**Results:**

Penetrating keratoplasty (PK) accounted for 52.4% of procedures, Descemet membrane endothelial keratoplasty (DMEK) for 42.9%, and deep anterior lamellar keratoplasty (DALK) for 4.7%. The main indications were bullous keratopathy (30.47%) and FECD (30.04%). Endothelial disease without significant subepithelial fibrosis supported DMEK, while stromal pathology with a functional endothelium favored DALK. Deep stromal fibrosis, corneal perforation, or extensive infection more frequently required PK.

**Discussion:**

*The findings suggest that the extent and depth of corneal involvement are key determinants of surgical technique selection. Advanced stromal or subepithelial fibrosis limited the benefit of endothelial keratoplasty,* highlighting an important limitation of selective lamellar techniques in advanced corneal disease.

**Conclusions:**

*Integration of clinical, histopathological, and imaging data helps identify the affected corneal layer and supports individualized selection of the corneal transplantation technique.*

## Introduction

Corneal transplantation has evolved significantly over the past decades, shifting from conventional full-thickness corneal replacement techniques (penetrating keratoplasty) toward selective lamellar procedures tailored to the affected corneal layer [1]. This transition has been driven by advances in surgical techniques, a better understanding of corneal disease pathophysiology, and the development of high-resolution imaging modalities [2,3]. Currently, the choice of corneal transplantation technique depends on the lesion’s location and extent, aiming to preserve as much healthy tissue as possible, reduce graft rejection risk, and optimize postoperative visual outcomes [4-6].

Histopathological examination plays an essential role in establishing a definitive diagnosis by identifying structural changes characteristic of different corneal diseases and providing valuable insights into the underlying pathogenic mechanisms [7,8]. In parallel, anterior segment optical coherence tomography (AS-OCT) enables non-invasive, real-time assessment of corneal thickness, reflectivity, and structural integrity, facilitating preoperative planning and postoperative monitoring [9-12].

This study aimed to describe the distribution of corneal transplantation procedures and indications in a tertiary referral center and to analyze the main clinical, histopathological, and imaging correlations relevant to selecting the optimal surgical technique. In addition, the study aims to highlight the complementary role of histopathological and imaging assessment in guiding therapeutic decision-making and improving patients’ visual prognosis.

## Materials and methods

We conducted a descriptive analysis of the database, including 233 corneal transplants performed between January 2021 and January 2026. The type of surgical procedure, including **penetrating keratoplasty (PK), Descemet membrane endothelial keratoplasty (DMEK), and deep anterior lamellar keratoplasty (DALK),** as well as the underlying pathology representing the surgical indication, were recorded. Results were expressed as absolute frequencies and percentages.

For representative cases of pseudophakic bullous keratopathy, Fuchs endothelial corneal dystrophy (FECD), keratoconus, and fungal keratitis, slit-lamp biomicroscopy, AS-OCT, and hematoxylin and eosin-stained histopathological sections were analyzed. The study was conducted at the “Prof. Dr. Mircea Olteanu” Clinical Emergency Eye Hospital, Bucharest, Romania.

## Results

Of the 233 procedures, 122 were PK (52.4%), 100 were DMEK (42.9%), and 11 were DALK (4.7%). Bullous keratopathy accounted for 71 cases (30.47%), while FECD accounted for 70 cases (30.04%). Infectious corneal disease, other corneal dystrophies, and keratoconus each accounted for 14 cases (6.0%) (**Table 1**).

**Table 1:** Distribution of corneal transplant indications and surgical techniques in the study cohort

Pathology	N (%)	DALK (%)	DMEK (%)	PK (%)
Bullous keratopathy	71 (30.47%)	0.0	28.0	35.2
Fuchs endothelial corneal dystrophy	70 (30.04%)	0.0	67.0	2.5
Infectious corneal disease	14 (6%)	27.3	0.0	9.0
Other corneal dystrophies	14 (6%)	18.2	0.0	9.8
Keratoconus	14 (6%)	36.4	0.0	8.2
Trauma	10 (4.29%)	0.0	0.0	8.2
Graft failure	10 (4.29%)	0.0	2.0	6.6
Corneal decompensation secondary to glaucoma	10 (4.29%)	0.0	3.0	5.7
Graft rejection	8 (3.43%)	0.0	0.0	6.6
Herpetic keratitis	6 (2.57%)	9.1	0.0	4.1
Corneal perforations	2 (0.85%)	0.0	0.0	1.6
Neurotrophic keratopathy	2 (0.85%)	9.1	0.0	0.8
Rheumatoid arthritis	1 (0.42%)	0.0	0.0	0.8
Recurrent corneal dystrophy	1 (0.42%)	0.0	0.0	0.8

We further analyzed correlations between clinical, histopathological, and imaging findings and the type of keratoplasty performed in pseudophakic bullous keratopathy, FECD, keratoconus, and fungal keratitis.

In patients with pseudophakic bullous keratopathy in the study cohort, diffuse corneal edema, stromal opacification, and Descemet membrane folds (**Fig. 1A**) were associated with stromal edema and disruption of stromal architecture on histopathological examination (**Fig. 1B**). AS-OCT showed increased corneal thickness and stromal hyperreflectivity (**Fig. 1C**), in agreement with the clinical and histopathological findings. In the absence of extensive fibrosis, the clinical and paraclinical data supported DMEK, with postoperative improvement in imaging appearance (**Fig. 1D**).

**Fig. 1:**
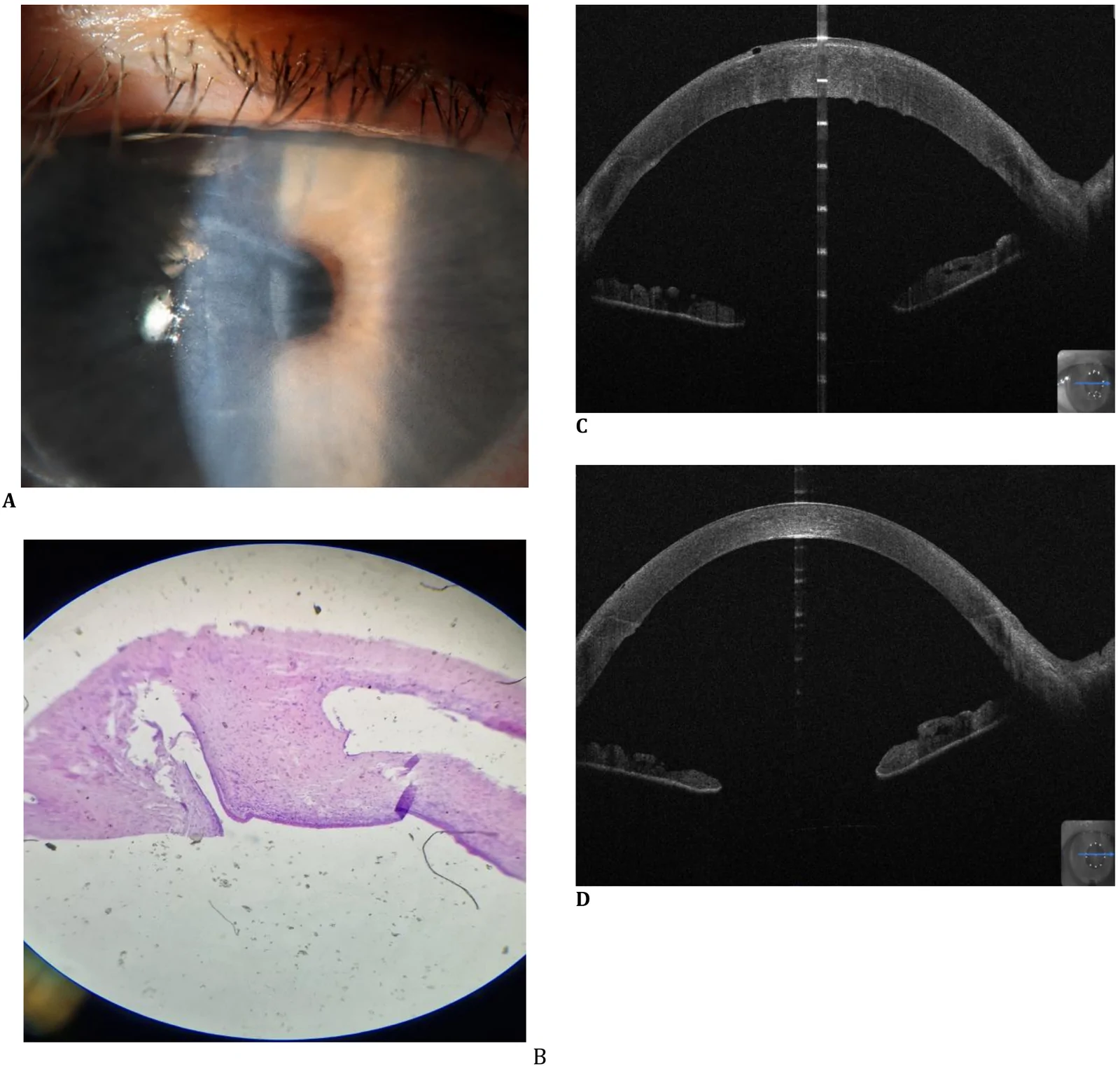
Representative case of bullous keratopathy: (**A**) clinical appearance; (**B**) histopathological appearance; (**C**) preoperative AS-OCT; (**D**) postoperative AS-OCT after DMEK. Images courtesy of the personal archive of Dr. Gheorghe.

In FECD, clinically identified corneal guttae (**Fig. 2A**) corresponded histopathologically to guttae on the posterior surface of the Descemet membrane. Thickening of the Descemet membrane, secondary to the deposition of amorphous and fibrillar extracellular material, was associated with reduced endothelial cell density (**Fig. 2B**). AS-OCT demonstrated irregularity of the Descemet membrane-endothelium complex and, in advanced stages, subepithelial fibrosis (**Fig. 3A**). Predominant endothelial involvement favored DMEK. In contrast, significant fibrosis limited its benefit (**Fig. 3B**).

**Fig. 2:**
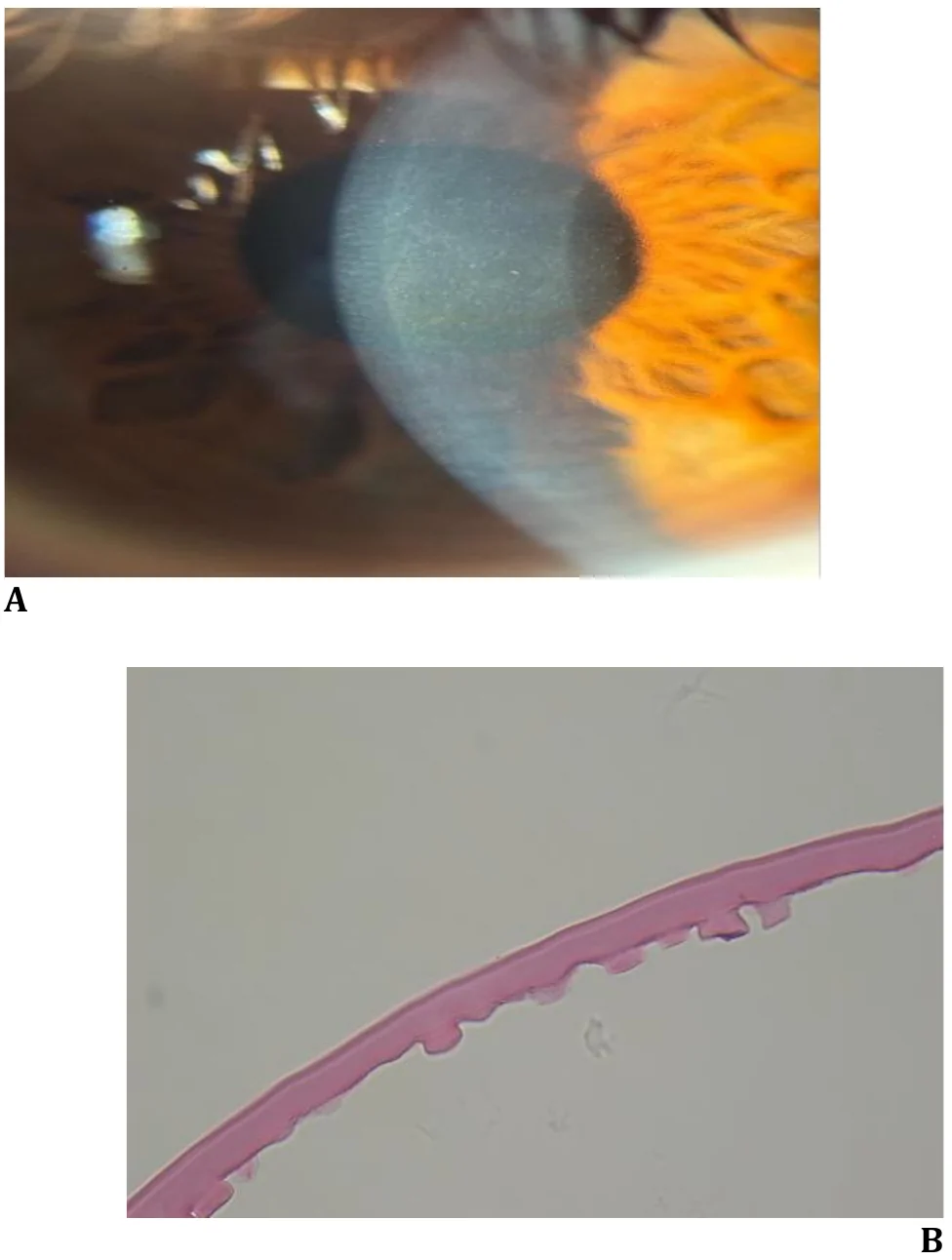
Representative case of a patient with FECD: (**A**) clinical appearance; (**B**) histopathological appearance. Images courtesy of the personal archive of Dr. Gheorghe.

**Fig. 3:**
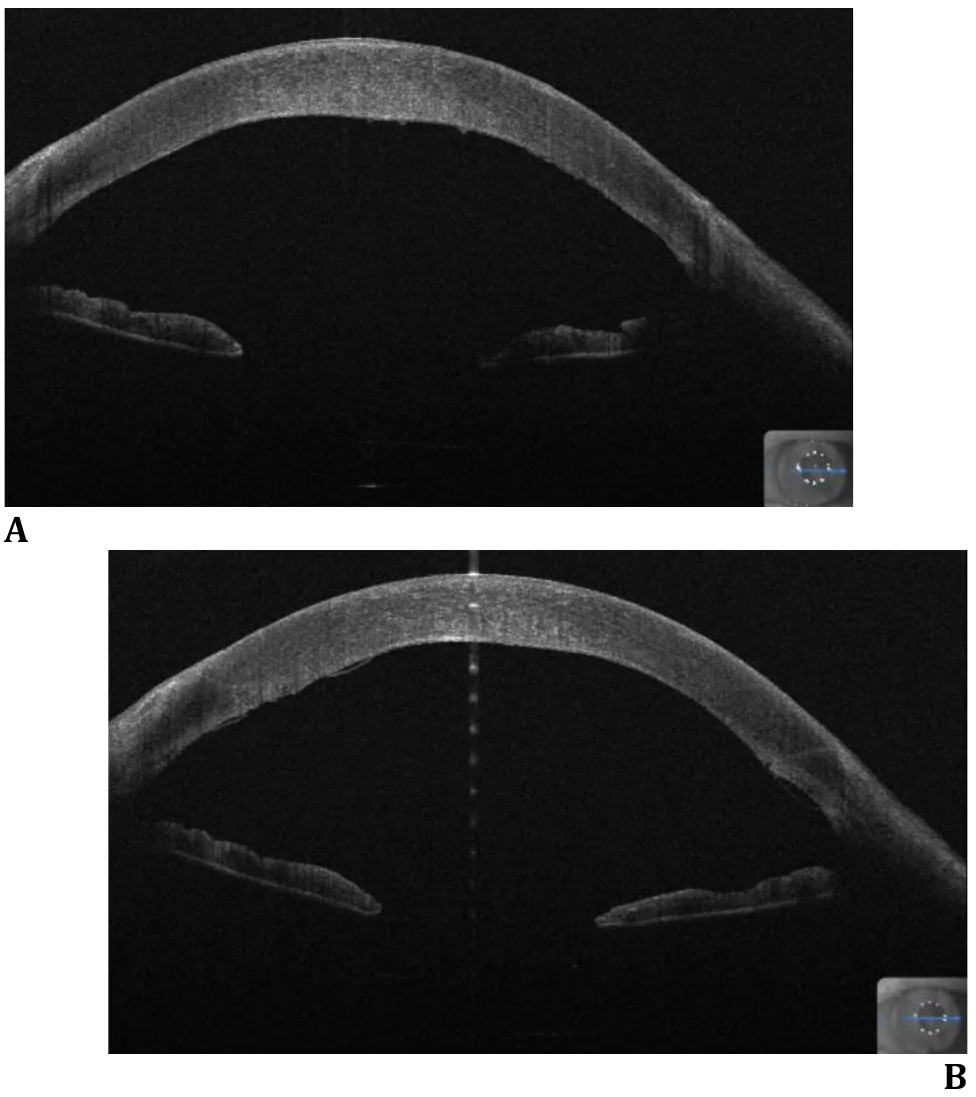
Preoperative AS-OCT and postoperative AS-OCT after DMEK. (**A**) AS-OCT showing increased corneal thickness, diffuse stromal hyperreflectivity suggestive of corneal edema, and subepithelial and stromal fibrosis. (**B**) After DMEK, the graft remained attached; however, corneal edema and fibrosis persisted, indicating the need for PK. Images courtesy of the personal archive of Dr. Gheorghe.

In keratoconus, ectasia, corneal thinning, hydrops, and post-hydrops scarring (**Fig. 4A**) correlated with epithelial irregularity, stromal disorganization, and stromal fibrosis (**Fig. 4B**). AS-OCT demonstrated corneal thinning and post-hydrops scarring (**Fig. 4C**). Anterior corneal lesions with a functional endothelium supported the indication for DALK, whereas post-hydrops corneal scarring required PK (**Fig. 4D**).

**Fig. 4:**
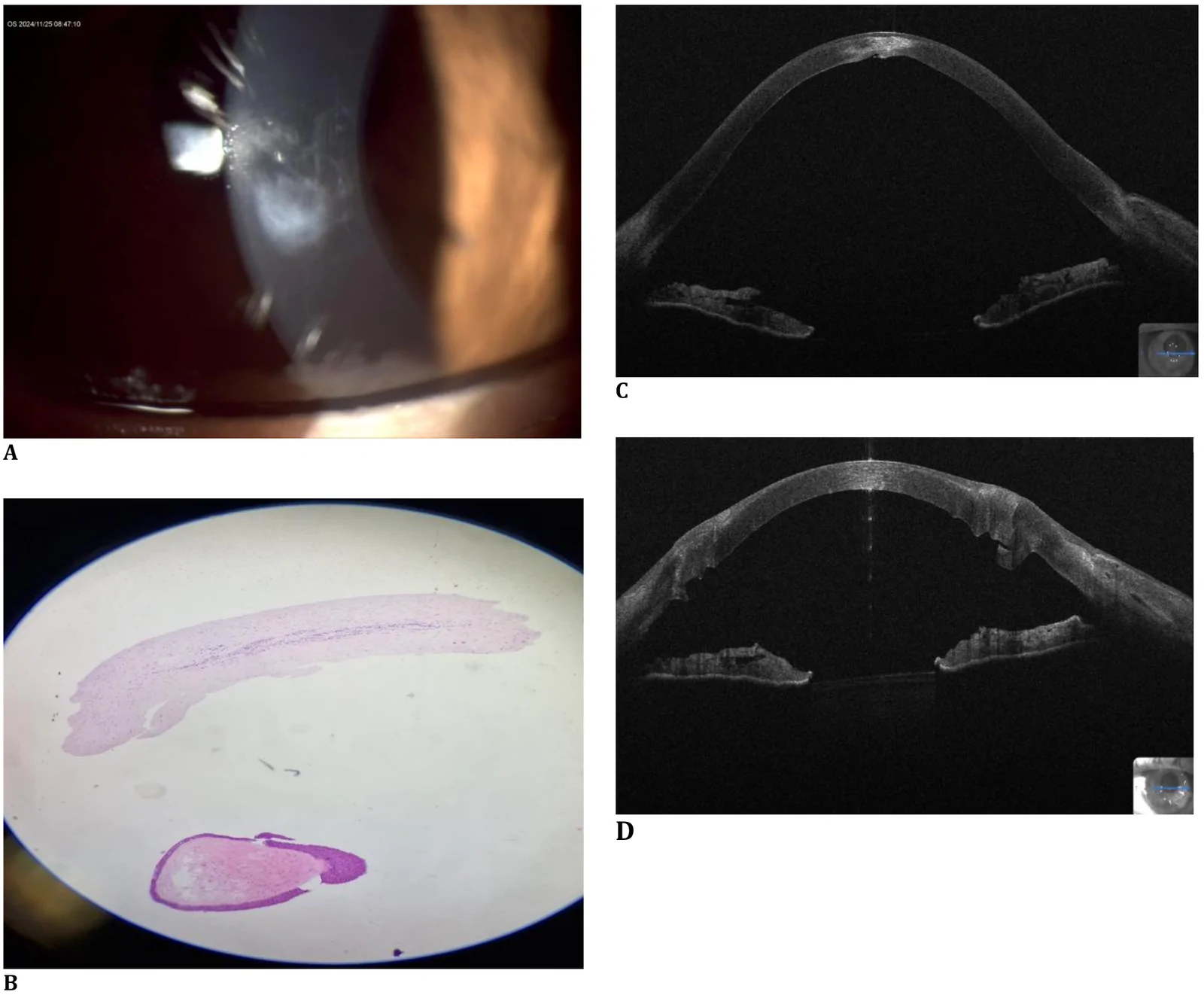
Clinical, histopathological, and imaging findings in keratoconus: (**A**) clinical appearance; (**B**) histopathological appearance; (**C**) preoperative AS-OCT; (**D**) postoperative AS-OCT after PK. The corneal graft is well positioned, with restored corneal architecture and replacement of opaque corneal tissue. At the graft-host junction, variations in corneal thickness and posterior surface irregularities are present, findings commonly observed postoperatively. Images courtesy of the personal archive of Dr. Gheorghe.

In fungal keratitis, the stromal infiltrate, ulcer with feathery margins, and corneal edema observed clinically correlated histopathologically with an inflammatory infiltrate and elongated, septate structures diffusely distributed throughout the full thickness of the stroma, findings suggestive of fungal invasion of corneal tissue (**Fig. 5**). AS-OCT may reveal increased corneal thickness, diffuse stromal hyperreflectivity corresponding to the inflammatory infiltrate, posterior shadowing caused by signal attenuation from dense infiltrates, and the presence of hyperreflective endothelial inflammatory plaques (**Fig. 6A**). Anterior infiltrates allowed a lamellar approach. In contrast, deep extension, endothelial involvement, or corneal perforation required therapeutic PK (**Fig. 6B**).

**Fig. 5:**
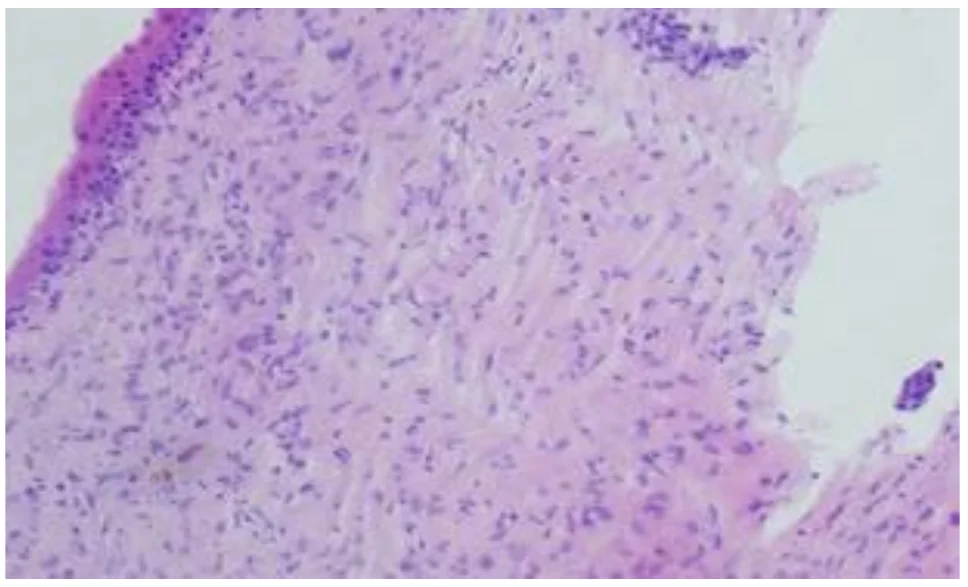
Histopathological findings in fungal keratitis. Image courtesy of the personal archive of Dr. Gheorghe.

**Fig. 6:**
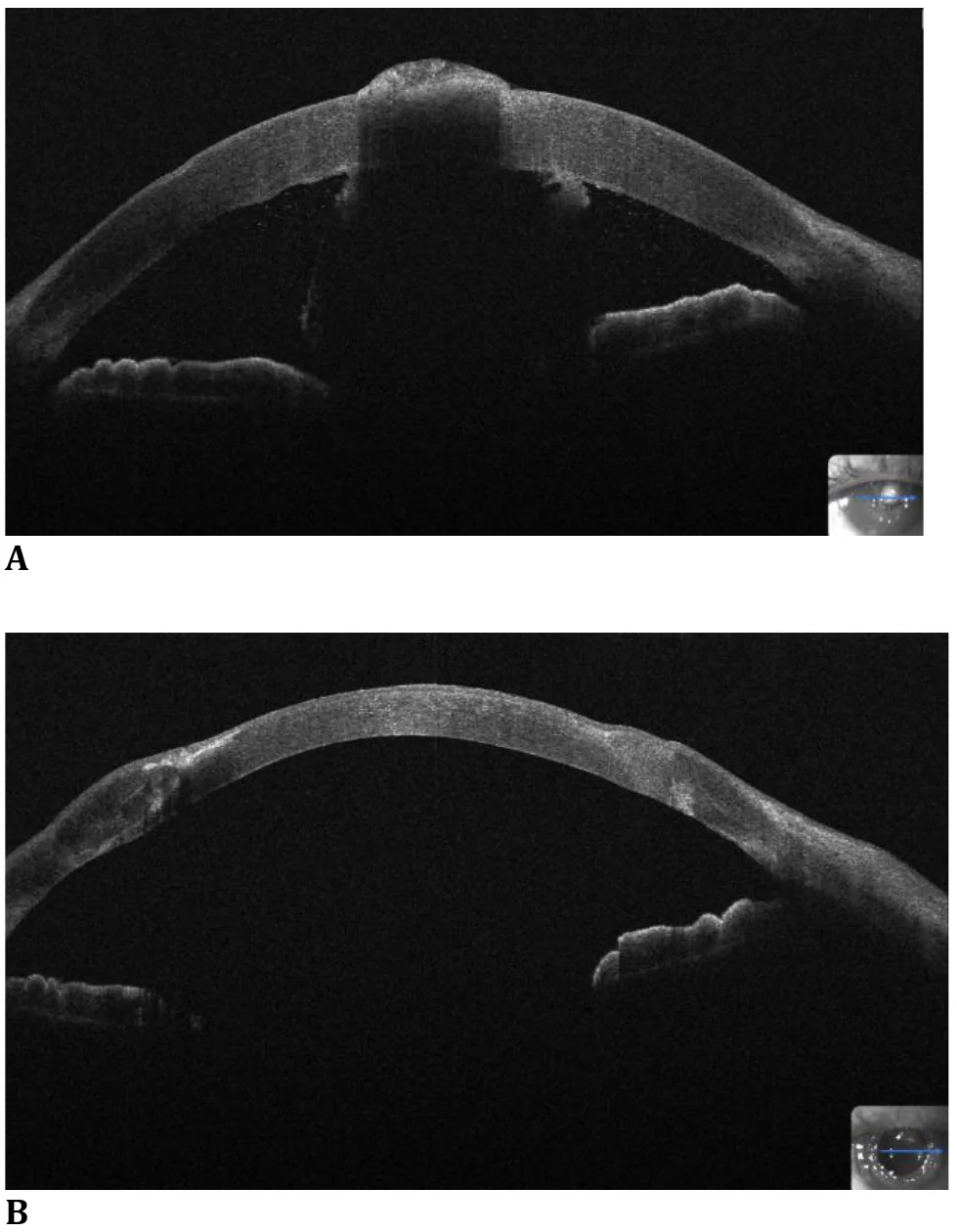
AS-OCT findings in fungal keratitis: (**A**) preoperative appearance; (**B**) postoperative appearance after therapeutic PK (**PK à chaud**). Images courtesy of the personal archive of Dr. Gheorghe.

## Discussion

In the study cohort, the main indications for corneal transplantation were bullous keratopathy and FECD, the latter representing one of the leading indications for corneal transplantation worldwide [13]. Other studies have reported stromal dystrophies or keratoconus as important indications for keratoplasty [14,15]. These differences may reflect the characteristics of the studied population and the center where the procedures were performed.

In endothelial diseases, assessing the severity of corneal changes played an important role in selecting surgical techniques. In the absence of extensive corneal fibrosis, predominantly endothelial involvement supported DMEK, whereas advanced stromal or subepithelial fibrosis limited the benefit of endothelial keratoplasty and favored PK. The literature also highlights the utility of AS-OCT both for identifying advanced structural changes in FECD and for postoperative assessment of graft attachment after DMEK [13].

In keratoconus, correlating clinical findings with histopathological and imaging features allowed differentiation between cases suitable for DALK and advanced forms with post-hydrops scarring that required PK. Current evidence describes keratoconus as a complex disorder whose pathogenesis also involves inflammatory mechanisms [16-19]. AS-OCT is useful in cases complicated by corneal hydrops, allowing assessment of Descemet membrane rupture and detachment [20,21].

In fungal keratitis, AS-OCT complements clinical and histopathological evaluation by assessing infiltrate depth and stromal changes [22]. Infiltrate hyperreflectivity correlates with its density on slit-lamp biomicroscopy, while pachymetric changes should be interpreted in the clinical context, as progressive corneal thinning may precede corneal perforation [20].

The analysis is descriptive and, in its current form, does not include demographic data, visual outcomes, or standardized quantitative parameters for the entire cohort. The correlations presented are illustrative rather than statistical correlation coefficients. A future study using predefined criteria and pre- and postoperative outcomes could assess the predictive value of clinical, histopathological, and imaging parameters.

## Conclusions

In the study cohort, PK was the most frequently performed procedure, while endothelial diseases represented the main indications for corneal transplantation. Integration of clinical examination with AS-OCT and histopathological findings supported DMEK for predominantly endothelial disease without significant fibrosis, DALK for stromal disease with a functional endothelium, and PK for deep lesions, advanced fibrosis, corneal perforation, or extensive infection.
